# High Energy Particle Radiation-associated Oncogenic Transformation in Normal Mice: Insight into the Connection between Activation of Oncotargets and Oncogene Addiction

**DOI:** 10.1038/srep37623

**Published:** 2016-11-23

**Authors:** Natarajan Aravindan, Sheeja Aravindan, Krishnan Manickam, Mohan Natarajan

**Affiliations:** 1 Department of Radiation Oncology, University of Oklahoma Health Sciences Center, Oklahoma City, OK, USA; 2 Stephenson Cancer Center, Oklahoma City, OK, USA; 3 Department of Pathology, University of Texas Health Science Center at San Antonio, San Antonio, TX, USA

## Abstract

Concerns on high-energy particle radiation-induced tumorigenic transformation of normal tissue in astronauts, and in cancer patients undergoing radiotherapy, emphasizes the significance of elucidating the mechanisms involved in radiogenic transformation processes. Mostly used genetically modified or tumor-prone models are less reliable in determining human health risk in space or protracted post-treatment normal tissue toxicity. Here, in wild type C57BL/6 mice, we related the deregulation of distinctive set of tissue-specific oncotargets in major organs upon ^56^Fe (600 MeV/amu; 0.5 Gy/min; 0.8 Gy) particle radiation and compared the response with low LET γ-radiation (^137^Cs; 0.5 Gy/min; 2 Gy). One of the novel findings is the ‘tissue-independent’ activation of TAL2 upon high-energy radiation, and thus qualifies TAL2 as a potential biomarker for particle and other qualities of radiation. Heightened expression of TAL2 gene transcript, which sustained over four weeks post-irradiation foster the concept of oncogene addiction signaling in radiogenic transformation. The positive/negative expression of other selected oncotargets that expresses tissue-dependent manner indicated their role as a secondary driving force that addresses the diversity of tissue-dependent characteristics of tumorigenesis. This study, while reporting novel findings on radiogenic transformation of normal tissue when exposed to particle radiation, it also provides a platform for further investigation into different radiation quality, LET and dose/dose rate effect in healthy organs.

The consequences of space radiation exposures and microgravity remain the primary health concerns for astronauts during deep space missions. During long-term missions (e.g., the 22–32 months estimated for Mars missions), astronauts may be exposed to high total doses (∼0.4–0.5 Gy) of galactic cosmic rays^1^. Armed with internal radiation (∼0.66 ± 0.12 Sv) data from the Curiosity Rover in transit to Mars^2^, researchers have estimated that each cell in an astronaut’s body would be traversed by a proton every three days, a helium nucleus every few weeks, and high charge and energy nuclei (e.g., ^12^C, ^16^O, ^28^Si, ^56^Fe) every few months during deep space missions^3^. In addition, the exploration-class space mission to Mars astronauts, most of whom are middle-aged, without any access to health care services for at least 2 years^1^,^4^, further raises the significance of risk estimation for cancer development. Considering the earth-based benefits, particle radiation has momentous importance for cancer treatment.

Studies focused on ground-based accelerator facilities^5^,^6^,^7^, which approximate the space radiation environment, have been conducted to better characterize radiation carcinogenic risk in long-term space travel. Although only 1% of galactic cosmic rays are composed of ions heavier than helium, almost half of the ionizing radiation dose-equivalent is predicted to be HZE particle-derived, with 13% from ^56^Fe particles alone. High-energy ions are of particular interest because of their dense track of ionization. A dense track structure results in high relative biological effectiveness (RBE)^8^, which is associated with concentrated, poorly repairable damage within organisms^9^. *In vitro* transformation studies have demonstrated a strong dependence on track structure and LET^10^,^11^. This was confirmed with more recent results showing dose-response consistency for induction of malignant and benign tumors using an iron ion beam.

*In vivo* models of cancer risk that sufficiently define the biology of tumor initiation or progression from HZE nuclei have been developed. Carcinogenesis studies in rat skin, mouse and rat mammary glands, and mouse models of leukemia and Hardarian gland tumor, showed a striking effectiveness for charged particles in cancer initiation and a strong dependence on linear energy transfer (LET)^12^. This was further supported by reports on particle radiation-induced mouse tumorigenesis in liver, gastrointestinal tract and lung^13^,^14^,^15^,^16^. Since most of the studies relied on cancer-prone or genetically modified animal models to overcome the long latency period, the evidence of cancer onset or progression upon high LET radiation is unclear when interpreted for similar incidence with middle-aged, healthy astronauts. In addition, the responses of organ sites that are differentially susceptible to prolonged low doses of high LET radiation during whole body irradiation are concealed when organ-specific tumor-susceptible animal models are used. Carcinogenic risk estimation with wild-type animal models is likewise challenging, due to the minimal chance of developing detectable tumors in the animals’ lifetimes.

The alterations in the architectural network of the homeostatic cellular signaling mechanism by modifying the functional aspects of signaling mediators and fixation of those alterations are known to translate into cancer onset. These events are believed to be preceded by activation of oncotargets that are responsible for cellular acquisition of oncogene addiction^17^. Repressed genes are equally important, since some tumors require a chronic suppression of critical oncogene(s) upon which tumorigenesis and progression depend. Inhibition of that single mediator can achieve remarkable tumor regression^17^. Sometimes the addiction is not restricted to single genes^18^, as the oncogenic addiction signaling pathway is dependent on a set of complementing addictive genes in most tumors.

The concept of the oncogene addiction pathway was convincingly demonstrated in tissue culture systems, *in vivo* studies, and, to some extent, in clinical settings^17^,^18^,^19^. Its significance, to our knowledge, has not yet been examined in the context of radiation-induced cancer initiation and/or progression. When the footprints of oncotarget activation that favor oncogenic addiction signaling pathways are detectable, this will allow us to predict the protracted cancer risk. Therefore, we must define whether particle radiation deposits energy to activate oncotargets that favor specific oncogenic addiction pathways. Oncogenic addiction pathways may, independently or in association with a non-oncogenic addiction route, orchestrate the initiation of protracted carcinogenic processes. Unraveling the occurrence of this architectural network in normal mice may significantly contribute to cancer risk assessment for normal healthy astronauts. We also seek to understand the extent of normal tissue toxicity in both particle radiation therapy and conventional treatment.

Here, we investigated the unexplored influence of particle radiation exposure on the onset of primary carcinogenic events after approximated space radiation from a ground-based accelerator in a manifold of healthy tissues from a wild-type C57BL/6 mouse model. Mice exposed to iron ions at 600 MeV/nucleon at a total dose of 0.8 Gy were examined for the activation of mediators in major organ sites, including the brain, gut, kidney, liver, lungs, spleen, and small and large intestine. The present study identified unique transcriptional rearrangements of 88 custom-archived oncotargets (in contrast to low LET radiation associated response), their translational modifications, changes in apoptotic echelons that drive selection pressure, deregulation of matrix proteins and early epithelial differentiation proteins, and genetic instability. For the first time, we used the concept of oncotarget-driven oncogene addiction to understand the radiogenic neoplastic transformation processes in normal tissues, contributing significantly to cancer risk assessment for astronauts and defining the extent of normal tissue toxicity in particle radiation therapy.

## Methods

### Animal experiments and irradiation procedures

All animal procedures were performed in accordance with National Institutes of Health guidelines under a protocol approved by the Animal Care and Use Committee, University of Texas Health Science Center at San Antonio, Texas. The animals were housed in autoclaved cages with bedding materials in a designated room with a 12-h dark and light cycle maintained at 22 °C in 50% humidity. All animals were provided with certified rodent diet and filtered water *ad libitum*. For the selected doses of high linear energy transfer (high-LET) radiation exposures, 10-week-old C57BL/6 J wild-type mice (*n* = 8/group; Jackson Laboratory, Bar Harbor, ME) were transported to the NASA Space Radiation Research Facility (NSRL) and individually loaded into animal restraining cages. Mice were exposed to 0.8 Gy of ^56^Fe (600 MeV; LET 181 keV/μm) at an average dose rate of 50 cGy/min at room temperature (22 °C). For low LET radiation exposures, mice were exposed to ^137^Cs gamma rays to a total dose of 2 Gy and at a dose rate of 50 cGy/min (Atomic Energy of Canada Ltd, Montreal, Canada) in a ventilated mouse pie cage (Braintree, Inc., MA). Animals were euthanized in accordance with the American Veterinary Medical Association (AVMA) panel of euthanasia guidelines, i.e., by an overdose of vaporized isoflurane (>3%) followed by cervical dislocation. Tissues were harvested and were immediately placed in RNA*later*^*®*^ stabilization solution (ThermoFisher Scientific, Inc.) for RNA preparation and QPCR profiling. Tissues were fixed in 10% neutral buffered formalin for tissue processing and immunohistochemical procedures or snap-frozen in liquid nitrogen and stored at −80 °C for protein analysis and other measures of molecular endpoints.

### Quantitative oncogene transcriptome profiling

Total RNA extraction and real-time QPCR profiling were performed at 30 days post-exposure as described earlier^20^,^21^. We used custom-made transcriptome profilers (www.Realtimeprimers.com) pertaining to interacting functional networks of oncogene addiction. We archived a unique oncogene profile (Table S1) and constructed the QPCR profiler in collaboration with realtimeprimers.com. We used this highly selected QPCR profiler instead of an all-encompassing gene array because the profiler provides a well-characterized outline of the selected genes governing the cellular signaling mechanism that directs the initiation and fixation of carcinogenic processes. The ΔΔ^ct^ values calculated by normalizing the gene expression levels to those of the housekeeping genes were then compared between groups. The relative expression level of each gene was expressed as a fold change. Group-wise comparisons were made using GraphPad PRISM to compare the gene loss or gain after high-LET radiation in the manifold of healthy tissues.

### QPCR

We used real-time QPCR to analyze the transcriptional regulation of *Tal2, MYCN, nRAS, MYC* and *kRAS,* in brain, spleen, small intestine (SI), lung, liver, kidney and gut tissue of mice exposed to high-LET (0.8 Gy of ^56^Fe, 600 MeV; LET 181 keV/μm) or low LET (2 Gy of ^137^Cs gamma rays) radiation as described in our earlier studies^22^,^23^. We used β-actin as a positive control, and a negative control without template RNA was also included. The ∆∆^Ct^ values were calculated by normalizing the gene expression levels to β-actin, and the relative expression level was expressed as a fold change. Group-wise comparisons were made using analysis of variance with Tukey’s post-hoc correction. Further, the expression patterns of these genes in various tissues between the QPCR profiling and individual gene QPCR were compared using correlation analysis (GraphPad PRISM).

### Tissue microarray (TMA) construction, high-content confocal immunofluorescence (IF), and automated immunohistochemistry (IHC)

All TMA construction procedures were performed in the Stephenson Cancer Center (SCC) - Cancer Tissue Pathology Core, as described earlier^24^,^25^. For this study, TMA was constructed with brain, liver, kidney, lungs, spleen, and gut tissue from mock-irradiated control mice (*n* = 4) and mice that were exposed to ^56^Fe ion radiation (*n* = 8). Individual tissue cores (1 mm) were obtained from 10% NBF-fixed and paraffin-embedded tissue blocks. All hematoxylin & eosin (H&E)-stained slides were individually reviewed. The most representative areas were arrayed in the recipient paraffin block. Four-μm-thick multiple sections were cut from the TMA and baked for subsequent IF or IHC. High-content confocal IF analysis was performed as described in our earlier studies^26^. In brief, deparaffinized TMA sections were rehydrated, neutralized (0.25 M Tris-HCl), blocked (0.5% BSA-2% FBS), and labeled with primary antibody under humidified conditions. We examined the cellular localization and expression of *Fosb, JunD, Myb, nMYC, STAT3, TAL2,* and *VEGFα* in brains, livers, kidneys, lungs, spleens, and guts from mice that were either mock-irradiated (mock-IR) or exposed to whole body high-LET radiation. Appropriate tissue morphologic/pathologic (H&E) controls and negative (no primary antibody) controls were examined in parallel. The primary protein was tagged with secondary Ab tagged with Alexa Fluor-488^®^, while the cell membrane was marked with WGA-Alexa Fluor-594^®^ and nuclear counterstained with DAPI. Stained TMA were then subjected to high content IF confocal imaging with Operetta and were analyzed for cytoplasmic/nuclear/total positivity quantification with Operetta integrated Columbus image data analysis. Group-wise comparisons were performed using two-way ANOVA with Bonferroni’s post-hoc correction (GraphPad Prism).

Immunohistochemical staining was performed using Leica BOND-III fully automated staining system^®^ (Leica Biosystem Inc., Buffalo Grove, IL) as described in our earlier studies^26^,^27^. In the present study, we examined the cellular localization and expression of Casp3 (active form), Cytokeratin 5, SULF2, P63, Desmin, Decorin, and Col3 in brains, livers, kidneys, lungs, spleens, and guts from mice that were either mock-irradiated or exposed to whole body high-LET radiation. An appropriate tissue morphologic/pathologic (H&E) control and negative (no primary Ab) controls (data not shown) were examined in parallel. The slides were micro-digitally scanned using an Aperio Scanscope (Aperio Technologies, Inc., Buffalo Grove, IL) slide scanner, and the virtual slide was constructed with digital histology. This process allows for the assembly of tissue collections in a tissue microarray (TMA) with variable magnifications. The digital images were then analyzed using Aperio integrated Spectrum (Aperio) software, the web-based database system, and were then presented as percent control. Group-wise comparisons were performed using GraphPad Prism.

### Cell death (TUNEL) analysis

High-LET radiation associated with induced programmed cell death was assessed by IHC and quantified on a single-cell level based on labeling DNA strand breaks with terminal deoxynucleotidyl transferase (TUNEL Assay). All TUNEL assay procedures were performed on the customized TMA with brain, liver, kidney, lungs, spleen, and gut tissues archives from mock-IR and irradiated animals (constructed as discussed above) in the SCC-Tissue Pathology Core using a commercially available *In Situ cell death detection kit* (Roche Diagnostics, Mannheim, Germany). Appropriate positive controls were included with individual sections of corresponding tissue with induced DNA strand breaks with recombinant DNAse I treatment before TUNEL labeling. Corresponding individual tissue types without Tdt enzyme mix were included as negative controls. The slides were micro-digitally scanned using an Aperio Scanscope (Aperio Technologies, Inc., Buffalo Grove, IL, USA) slide scanner. The virtual slide with digital images was then analyzed using Aperio integrated Spectrum (Aperio). TUNEL positivity was quantified using Aperio TMA analysis. Group-wise comparisons were performed using GraphPad Prism.

### *In vivo* Micronucleus Assay

The micronucleus assays was performed on the customized TMA with the brain, liver, kidney, lung, spleen, and gut tissues archive from mock-IR and irradiated animals (constructed as discussed above). Automated H&E staining procedures were performed using a Multi-stainer (Leica). Slides were observed under a Nikon Eclipse Ni microscope equipped with a Nikon digital site DS-U3 camera under 1000X magnification. All micronuclei grading was performed in a double-blinded fashion. At least 1,000 cells per core were counted. The criteria for the micronuclei in the brain, kidney, liver, spleen, lungs, and gut tissues were uniform across the tissue types and included: i) the same staining as the main nucleus; ii) smaller than the diameter of the main nucleus, and; iii) not attached to the main nucleus.

## Results

### HZE ion radiation instigates oncogene addiction in healthy tissues

**Figure 1:**
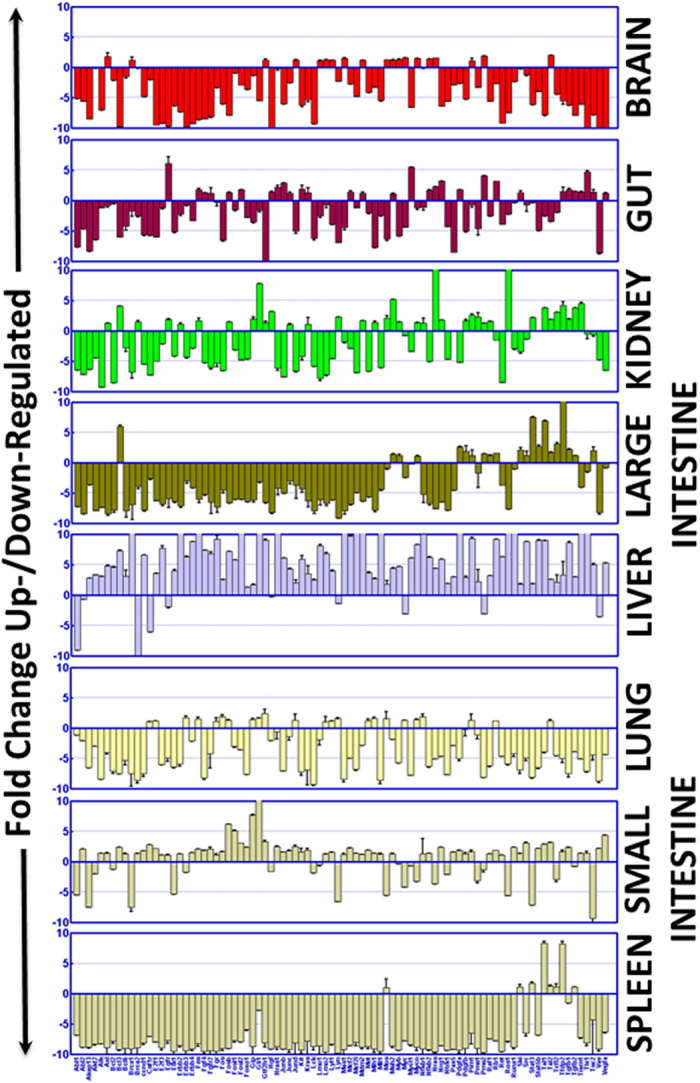
Histograms obtained from the quantitative QPCR profiling showing transcriptional modifications of 88 oncogenes in mouse brain, gut, kidney, liver, lung, spleen, and large and small intestine in response to HZE ^56^Fe particle radiation exposure. A custom-made transcriptome profiler with an archive of a unique oncogene profile pertaining to interacting functional networks of oncogene addiction that govern cellular signaling and drive the initiation of carcinogenic processes was used. Individual gene expression levels were background subtracted. The inter-profile variations are normalized with internal positive/housekeeping controls. The relative expression level of each gene is expressed as fold change compared with the mock-IR controls (mean and *SD*). Group-wise comparisons were made using GraphPad PRISM. For ease of comparison, all genes investigated, irrespective of their expression status, are included in the graph.

To define the high-energy particle radiation-associated oncogenic addiction in healthy tissues, we examined the oncogenic biomarker transcription profile after iron ion exposure (600 MeV/u; 0.8 Gy at 0.5 Gy/min) in major organ sites, including the brain, gut, kidney, large intestine, small intestine, liver, lung, and spleen. We used a mouse oncogene quantitative PCR profile (Realtimeprimers.com, Elkins Park, PA) that comprises the customized archive of 88 oncotargets and is equipped with eight internal positive and negative controls (Table S1). Exposing the animals to whole body high-LET radiation prompted a wide range of oncogene transcriptional responses in every tissue investigated. Compared with mock-irradiated controls, HZE ion radiation specifically activated transcription of **19** oncogenes, including *Axl, Brca1, Gtf2h1, Jund, Lmo1, Lmo2, Lyl1, Mas1, Mdm2, Mos, Msh2, Myb, Myc, Mycn, Nfkb2, Nras, Pim1, Pms2*, and *Tal2* in the mouse **brain** (Fig. 1). Likewise, high-LET radiation resulted in the activation of **30** oncogenes (*Egfr, Fes, Fgf4, Fgfr2, Fosb, Fosl2, Hgf, Hras1, Junb, Junc, Kit, Kras, Mcf2, Mdm2, Msh2, Mycl1, Nfkb2, Nras, Nrg1, Pdgfa, Pms2, Rb1, Ski, Tfdp2, Tgfb1, Tgfb2, Tiam1, Tlx1, Tsc2, Vegfa*) in the mouse **gut** (Fig. 1). Further, HZE ion radiation induced **37** genes (*Axl, Bcl3, Brca2, Egfr, Erbb2, Fes, Fosb, Gip, Gli1, Gtf2h1, Hgf, Junc, Kras, Lyn, Mdm2, Mlh1, Mos, Msh2, Myb, Mycn, Nfkb1, Nras, Nrg1, Pdgfb, Pim1, Pms1, Pms2, Raf1, Ros1, Stat3, Tal1, Tal2, Tcfl2, Tfdp2, Tgfb1, Tgfb2, Tiam1)* in **kidney** tissues (Fig. 1). In addition, we found transcriptional activation of ***21*** genes (*Bcl3, Msh2, Myb, Mycn, Pdgfa, Pdgfb, Pim1, Pms2, Raf1, Rb1, Ski, Src, Stat3, Stat5b, Tal1, Tal2, Tcfl2, Tfdp2, Tgfb1, Tgfb2, Tsc2*) in the **large intestine** and another **63** oncogenes (*Abl2, Alk, Axl, Bcl3, Bcl6, Brca2, ccnd1, Csf1r, E2f1, E2f3, Egfr, Erbb2, Erbb4, Fes, Fgf4, Fgfr2, Fgr, Fos, Fosb, Fosl1, Fosl2, Foxo1, Gip, Gli1, Gtf2h1, Hras1, Junb, Junc, Jund, Kit, Kras, Lmo2, Lyl1, Mas1, Mcf2, Mcf2l, Mdm2, Met, Mlh1, Mll1, Msh2, Nfkb1, Nfkb2, Nrg1, Pax5, Pdgfa, Pdgfb, Pim1, Raf1, Rb1, Ret, Runx1, Ski, Src, Stat5b, Tal1, Tal2, Tfdp2, Tgfb1, Tiam1, Tlx1, Vav, Vegfa*) in the **small intestine** tissues of mice exposed to iron ions (Fig. 1). Moreover, high-LET radiation led to the transcriptional activation of **8** (*Mos, Ski, Stat3, Tal1, Tal2, Tcfl2, Tfdp2, Tgfb2*) oncogenes in the **spleen** and another **23** oncogenes (*Csf1r, E2f1, Erbb3, Fes, Fgr, Fos, Fosb, Gip, Gli1, Gtf2h1, Jund, Lmo2, Lyl1, Lyn, Met, Mlh1, Mos, Myc, Mycn, Nfkb1, Pim1, Rb1, Tal2*) in **lung** tissues (Fig. 1). We observed a magnified oncogenic transcription response in **liver** tissues, with increased expression of **78** oncogenes after high-LET radiation (Fig. 1).

The unique tissue-specific gene expression pattern in response to high LET ^56^Fe radiation exposure is clearly apparent compared to the transcript expression pattern in mice exposed to low LET gamma radiation at a comparable dose equivalent. Same strain of mice, at the same age group exposed to ^137^Cs gamma rays to a total dose of 2 Gy at a dose rate of 0.5 Gy/min were compared with the high LET groups. In general, the relative biological effect (RBE) of low LET radiation is implicated as a factor of 1 through 4 depending on the endpoint analyzed and contingent on several other experimental samples and conditions, we chose a commonly used factor of around 2 to compare low LET at 2 Gy with ^56^Fe ion at 0.8 Gy. The pattern of microarray profiling is significantly different in low LET radiation compared to particle radiation exposure (Figure S1). Differing to the ^56^Fe ion response, we observed a largely inverse tissue response after whole body low-LET radiation. Low LET radiation exposure resulted in sustained activation of limited oncogenes in brain, gut, kidney, liver and small intestine (8, 6, 8, 6 and 21 genes respectively) (Figure S1), where we observed magnified oncogenic transcription response (19, 30, 37, 78 and 63 respectively) with high-LET radiation exposure. Conversely, we observed a heightened oncogenic transcription response in large intestine, lungs and spleen (42, 56 and 53 genes respectively) after low-LET radiation (Figure S1), while relatively marginal response was observed in these tissues (21, 23 and 8 respectively) after ^56^Fe ion exposure. Together, these data identify a distinctive oncogenic transcriptional response that sustains in mouse tissues upon ^56^Fe ion exposure.

**Figure 2:**
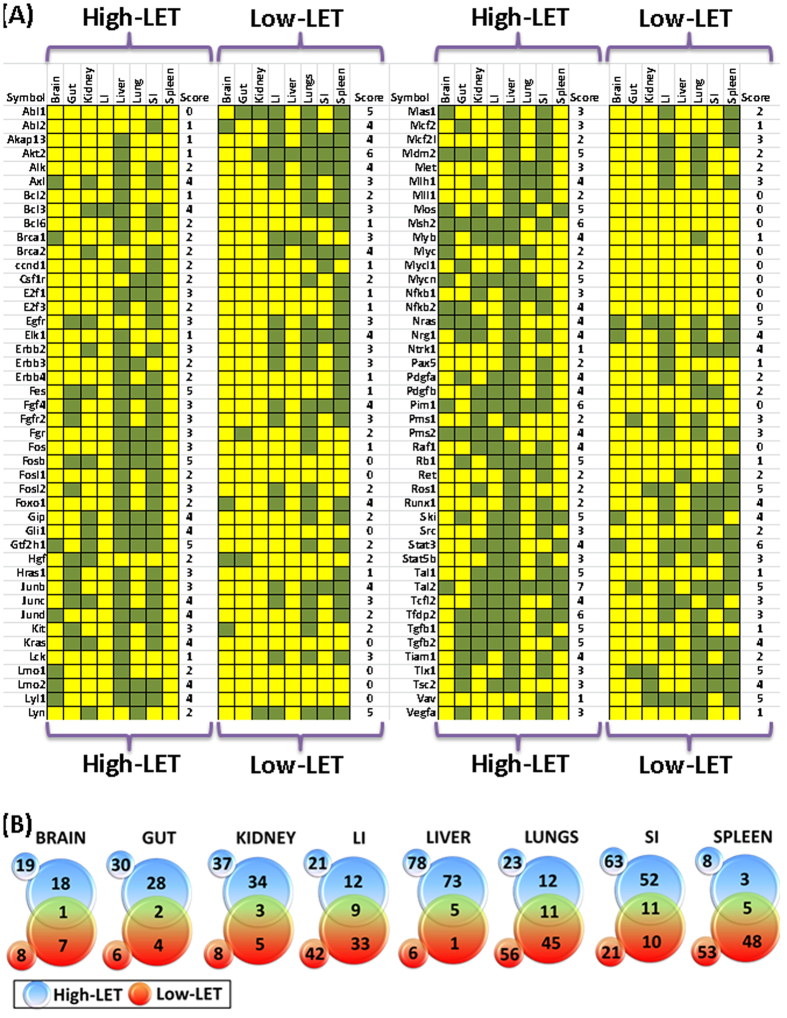
(**A**) Heat map showing the transcriptional expression status of each oncogene across the tissues investigated after HZE particle radiation or low-LET radiation, arranged in alphabetical order. The tissue scoring and expression of each oncogene in numerous tissues were also included. HZE particle radiation resulted in the heightened transcription of *TAL2* in seven of the 8 tissues investigated. Green: activated transcription; Yellow: non-activated or decreased transcription. (***B***) Venn diagrams constructed from High-LET: low-LET radiation crisscross analysis of activation sustained oncogenes in brain, gut, kidney, large intestine (LI), liver, lungs, small intestine (SI) and spleen showing exclusive HZE ion exposure response. *Numbers in small circles outside the Venn diagram *= *total number of oncogenes activated in response to high/Low-LET radiation; numbers in bigger circles* = *genes activated exclusively in response to high/low-LET radiation; numbers in overlapping region* = *genes activated in common between high- and low-LET radiation.*

Tissue comparison analysis after particle radiation exposure revealed no tissue-independent transcriptional activation of any oncogene either after HZE ion or low-LET radiation (Fig. 2A). However, *Tal2* showed near tissue-independent activation with increased levels in all tissues investigated, except mouse gut tissues with HZE ion. In addition, three genes, *Msh2, Pim1*, and *Tfdp2*, were increased at least in six of the eight tissue types investigated (Fig. 2A). A number of genes showed tissue-independent expression patterns, with 11 genes that were activated across at least 5 tissues, 21 genes activated across at least 4 tissues, 20 genes activated across at least 3 tissues, and 23 genes activated across at least 2 tissues after high-LET radiation exposure. Conversely, 8 genes showed tissue-dependent expression, with *ABL2* and *VAV* only in the small intestine and *AKAP13, AKT2, BCL2, ELK1, LCK*, and *NTRK1* only in liver tissues (Fig. 2A). Interestingly, one gene, *ABL1*, remained unaltered in all tissues after high-LET radiation exposure. In disparity, *AKT2* and *STAT3* showed near tissue-independent activation (at least in six tissues) with low-LET radiation. In addition, 7 genes, *Abl1, Lyn, Nras, Ros1, Tal2, Tlx1* and *Vav* were increased at least in five of the eight tissue types investigated (Fig. 2A). Also many genes showed tissue-independent expression patterns, with 14 genes that were activated across at least 4 tissues, 16 genes activated across at least 3 tissues, and 17 genes activated across at least 2 tissues, after low-LET radiation exposure (Fig. 2A). Conversely, 15 genes showed tissue-dependent expression and 17 genes remained unaltered in all tissues after low-LET radiation exposure (Fig. 2A).

**Figure 3:**
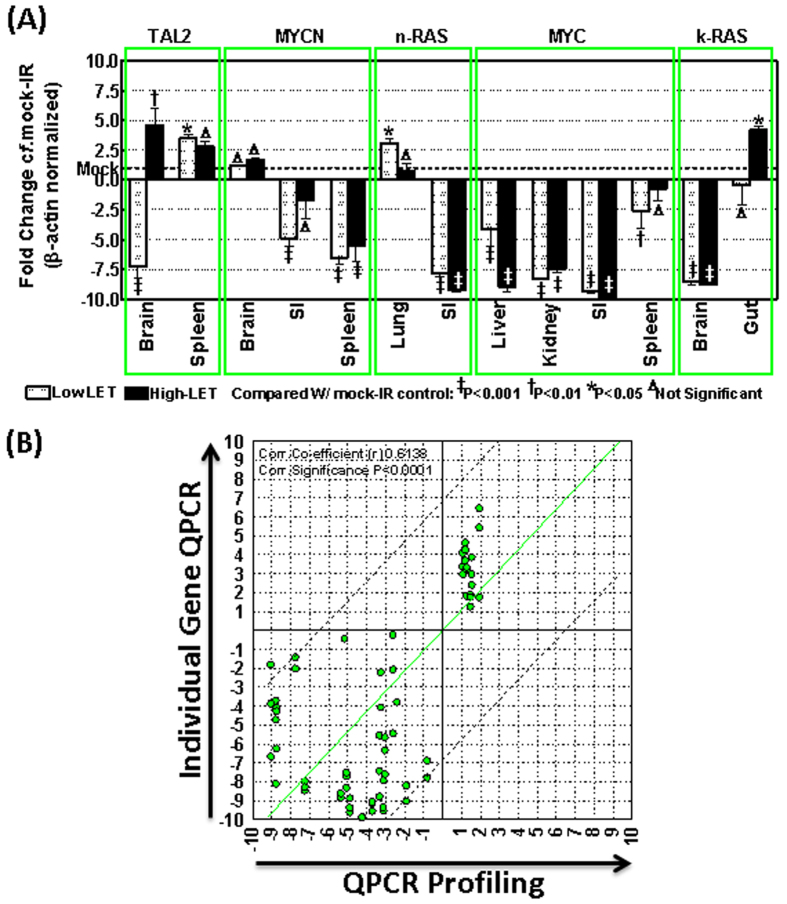
(**A**) Histograms obtained from individual gene QPCR analysis showing the altered *TAL2, MYCN, nRAS, MYC* and*, kRAS* mRNA levels in select tissues of mice exposed low-LET (2 Gy) or high-LET (0.8 Gy) radiation. β-actin normalized expression levels were computed for fold change (compared to mock-IR controls) and the mean and SD are plotted. (**B**) Gene expression comparison analysis (QPCR profiling vs. individual gene QPCR) computing the expression profiles of *TAL2, MYCN, nRAS, MYC* and *kRAS* in select tissues of mice exposed low-LET or high-LET radiation showing significant correlation between analytic platforms.

Further, high-LET vs low-LET crisscross expression profile analysis identified unique oncogenic transcriptional response after high-LET radiation in each tissue analyzed. For instance, of the 19 genes that sustained activation in brain tissue after HZE ion, 18 remained exclusive as HZE response (Fig. 2B). Like-wise, 28 of 30 genes in gut, 34 of 37 genes in kidney, 12 of 21 genes in large intestine, 73 of 78 genes in liver, 12 of 23 genes in lungs, 52 of 63 genes in small intestine and 3 of 8 genes in spleen showed select HZE ion exposure response (Fig. 2B). Similarly, we also observed a low-LET radiation select response in these tissues (7 of 8, brain; 4 of 6, gut; 5 of 8, kidney; 33 of 42, large intestine; 1 of 6, liver; 45 of 56, lungs; 10 of 21, small intestine and; 48 of 53, spleen) recognizing the unique radiation LET dependent oncogene addiction in healthy tissues (Fig. 2B). In addition, the sustained oncogenic transcriptional profile was validated with individual gene QPCR. For this, sustained activation of select genes (*Tal2, MYCN, nRAS, MYC, kRAS*) in select tissues of mice exposed to HZE ion or low-LET radiation were examined. The individual gene QPCR analysis of high-LET and low-LET radiation altered *Tal2* (in brain and spleen tissues), *MYCN* (in brain, small intestine and spleen), *nRAS* (in lung and small intestine), *MYC* (in liver, kidney, small intestine and spleen) and, *kRAS* (in brain and gut) demonstrated near-identical expression patterns to that of transcriptome profiling (Fig. 3A). Moreover, correlation analysis of the gene expression profiles between the profiling and QPCR demonstrated a significant (P < 0.001) correlation co-efficient, validating observed outcomes after HZE ion exposure with QPCR profiling (Fig. 3B). Quantitative profiling of the transcription of 88 oncogenes in this panel of eight tissues defined the blueprint of tissue-independent and tissue-dependent oncogenic response after high-LET radiation, and further identified crucial tissue specific targets that warrant in-depth investigations. Therefore, these results suggest that *TAL2* might serve as a universal marker to identify the instigation of an oncogenic response to high-LET radiation.

### HZE ion radiation increases JUNC, JUND, MYB, FOSb, MYCN, cMYC, STAT3, TAL2, and VEGFα expression and localization in healthy tissues

To further substantiate that high-LET radiation increases crucial and defined oncogenic players that functionally orchestrate carcinogenesis, we examined the translational modifications of TAL2 (activated transcription in 7 tissues), FOSb, MYCN (activated in 5 tissues), JUNC, JUND, MYB, STAT3 (activated in 4 tissues), VEGFα (activated in 3 tissues), and cMYC (activated in 2 tissues) in brain, liver, kidney, lung, spleen, and gut tissues (Figure S2). To avoid possible inconsistencies that normally occur in immunofluorescence (IF) staining across samples, the tissue microarray (TMA) was constructed with the manifold of tissues from mock-irradiated animals and animals exposed to high-LET radiation. The protein localizations were examined with high-content confocal immunofluorescence coupled with Columbus image (nuclear/cytoplasmic/membrane/total positivity) analysis. TMA cores were carefully constructed utilizing histopathological evaluations of individual H&E stained tumor tissues and were further confirmed with TMA and H&E staining.

**Figure 4:**
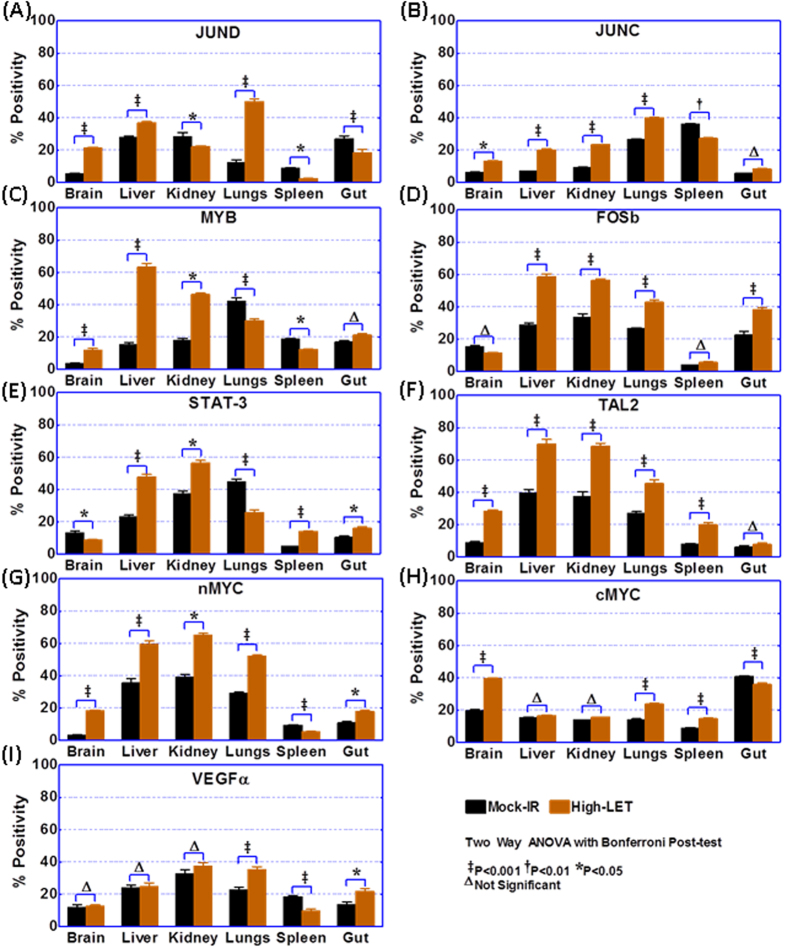
Histograms showing the expression levels of (**A**) *JUND, (**B**) JUNC, (**C**) MYB, (**D**) FOSb, (**E**) STAT3, (**F**) TAL2, (**G**) MYCN, (**H**) cMYC, and (**I**) VEGF*α in brain, liver, kidney, lung, spleen, and gut tissues from mice that were either mock-irradiated or exposed to whole body HZE particle radiation. Quantification of the corresponding cytoplasmic/nuclear/total positivity was performed using Columbus image data analysis from Operetta high content IF confocal imaging. The primary protein was tagged with secondary Ab tagged with Alexa Fluor-488^®^, while the cell membrane was marked with WGA-Alexa Fluor-594^®^ and nuclear counterstained with DAPI. Group-wise comparisons were performed in GraphPad Prism using two-way ANOVA with Bonferroni’s post-hoc correction.

**JunD** proto-oncogene IF staining revealed basal levels of positivity in mock-irradiated controls with relatively high expression in mouse liver and kidney and minimal-to-no expression in brain tissues (Figure S2). The positive staining of JunD protein was predominantly localized in the nucleus, often only in a fraction of the cells. Compared with the mock-irradiated controls, JunD immunoreactivity was intense (*P* < 0.001) in brains, livers, and lungs from mice exposed to high-LET radiation (Fig. 4A). We observed a relatively robust increase in lung tissues. No increased expression of JunD was observed in kidney, spleen, and gut tissues.

Likewise, proto-oncogene **cJun** IF staining showed relatively high basal expression in lungs and spleens from mock-irradiated controls (Fig. 4B, Figure S2). Conversely, brain and gut tissues exhibited very low basal levels of cJun expression. Compared with these controls, HZE particle radiation profoundly increased cJUN levels in mouse brain (*P* < 0.05), liver (*P* < 0.001), kidney (*P* < 0.001), and lung (*P* < 0.001) tissues (Fig. 4B). No significant increase in cJUN expression was observed in spleen or gut tissues after high-LET exposure. **MYB** (V-myb avian myeloblastosis viral oncogene homolog) IF exhibited strong nuclear immunoreactivity and some weak cytoplasmic positivity (Figure S2). While lung tissues showed high levels of basal MYB expression, brain tissues exhibited marginal-to-null MYB expression in mock-irradiated controls (Fig. 4C). We observed a significant (*P* < 0.001) increase in the expression of MYB protein in brain, liver, kidney, and lung tissues from animals exposed to high-LET radiation (Fig. 4C). Similarly, **FOSb** (FBJ murine osteosarcoma viral oncogene homolog B) exhibited high baseline expression in liver, kidney, and lung tissues, with strong nuclear positivity and some weak-to-moderate cytoplasmic staining (Fig. 4D, Figure S2). High-LET radiation exposure resulted in increased (*P* < 0.001) expression of FOSb in liver, kidney, lung, and gut tissues (Fig. 4D). FosB remained unchanged in brain and spleen tissues, with and without radiation exposure.

Further, **STAT-3** (Signal transducer and activator of transcription-3) IF staining showed ubiquitous cytoplasmic and nuclear expression at variable levels with very high expression levels in kidney and lung tissues, but minimal levels in the spleen (Fig. 4E, Figure S2). Compared with mock-IR control samples, radiation exposure resulted in robust increase of STAT3 in liver, spleen (*P* < 0.001), kidney, and gut (*P* < 0.01) tissues (Fig. 4E). T-cell acute lymphocytic leukemia 2 (**TAL2**), which exhibited high transcriptional activity in 7 out of 8 tissue types, displayed high basal levels in liver and kidney tissues, but low levels in the gut (Fig. 4F, Figure S2). TAL2 IF exhibited strong granular cytoplasmic positivity. High-LET radiation exposure resulted in the significant (*P* < 0.001) expression of TAL2 in brain, liver, kidney, lungs, and spleen, but not gut, tissues (Fig. 4F). This protein expression pattern was consistent with our transcriptional profiling data. IF analysis of **MYCN** (V-myc avian myelocytomatosis viral oncogene neuroblastoma derived homolog), which is predominantly localized in the nucleus, demonstrated significant increases in mouse brain (*P* < 0.001), liver (*P* < 0.001), kidney (*P* < 0.05), lung (*P* < 0.001), and gut (*P* < 0.05) tissues after radiation exposure (Figure S2 and Fig. 4G). Likewise, radiation exposure resulted in the significant (*P* < 0.001) activation of **cMYC** (V-myc avian myelocytomatosis viral oncogene homolog) in mouse brain, lung, and spleen tissues (Figure S2 and Fig. 4H). **VEGFα** (Vascular endothelial growth factor α), which showed ubiquitous cytoplasmic localization, was expressed at high levels in kidneys (Figure S2 and Fig. 4I). Radiation exposure resulted in increased expression of VEGFα in mouse lung (*P* < 0.001) and gut (*P* < 0.05) tissues.

### High-LET radiation prompts carcinogenic events in healthy tissues

#### (i) HZE particle radiation-associated apoptosis and increased expression of active Casp3

Apoptosis is considered an anti-carcinogenic process, due to its crucial role in eliminating genetically unstable cells that have suffered DNA damage^28^,^29^. Conversely, there is a growing recognition that apoptosis is involved in carcinogenesis through apoptosis-promoting oncogenes^30^,^31^,^32^ coupling with their hyper-proliferative activities, which could create selection pressure to overcome apoptosis, thus allowing cancer cells to become more malignant^33^. Researchers have reported that caspase-3, a central effector of apoptosis, facilitates chemical/radiation-induced genetic instability and carcinogenesis. It has been shown that a significant fraction of cells exposed to radiation can survive Casp3 activation; this sub-lethal activation of Casp3 promoted persistent DNA damage and oncogenic transformation^34^.

**Figure 5:**
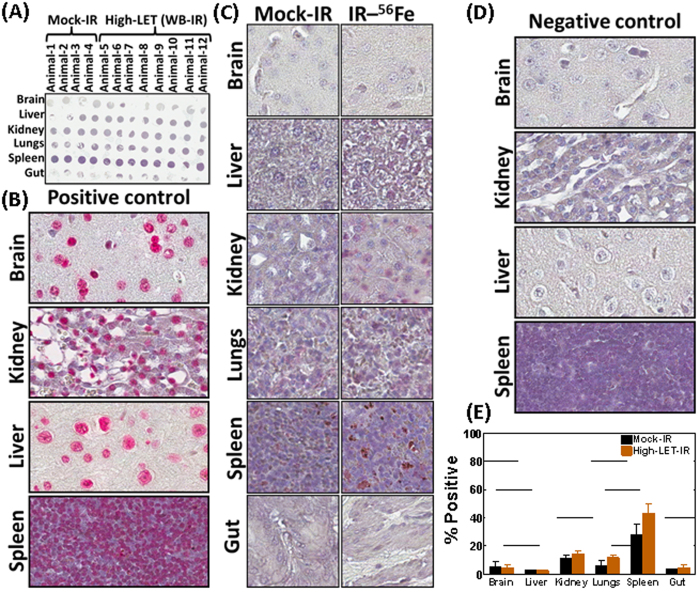
TUNEL staining analysis showing apoptotic modulations in mouse tissues exposed to HZE particle radiation. (**A**) Microphotograph of the TUNEL-stained TMA. A tissue microarray was constructed with the brain, liver, kidney, lugs, spleen, and gut tissues from the mock-irradiated mice or the mice exposed to HZE particle radiation. (**B**) Representative microphotographs of corresponding tissue TUNEL-positive controls. For induced TUNEL positivity DNA strand breaks were induced with recombinant DNAse I treatment. (**C**) Representative microphotographs showing comparative regions of TUNEL-positive cells in brain, liver, kidney, lugs, spleen, and gut tissues in mice exposed to mock-IR or HZE particle radiation. (**D**) Representative microphotographs of the corresponding TUNEL-negative tissue controls. For negative controls, TUNEL staining was performed without Tdt enzyme labeling. (**E**) Histograms obtained from TUNEL assay showing HZE-associated apoptotic alterations in HZE-exposed mouse tissues compared with mock-IR controls. TUNEL positivity was quantified using Aperio TMA analysis. Group-wise comparisons were performed using GraphPad Prism.

We first investigated the levels of induced apoptosis in healthy tissues after HZE particle radiation. A customized TMA constructed with brain, liver, kidney, lung, spleen, and gut tissues from mock-irradiated and irradiated animals was assessed for cell death using TUNEL assay (Fig. 5A). Corresponding individual tissue sections treated with DNase exhibited high levels of TUNEL-positive cells, and served as internal positive controls (Fig. 5B). Overall, we observed minimal baseline cell death in untreated mock-IR controls; visually, HZE particle radiation resulted in increased cell death to a certain degree (Fig. 5C). Terminal deoxynucleotidal transferase (Tdt) enzyme controls exhibited zero TUNEL-positive cells and served as the internal negative control (Fig. 5D). Quantification of the TUNEL-positive cells in tissues from mock-irradiated animals revealed minimal (brain, liver, gut), marginal (kidney, lungs), and relatively high (spleen) baseline apoptosis (Fig. 5E). HZE particle exposure resulted in insignificant cell death changes in mouse brain, liver and gut tissues. Although this finding was not statistically significant, we observed an elevated level of apoptosis in kidney, lung, and spleen tissues after high-LET radiation (Fig. 5E).

**Figure 6:**
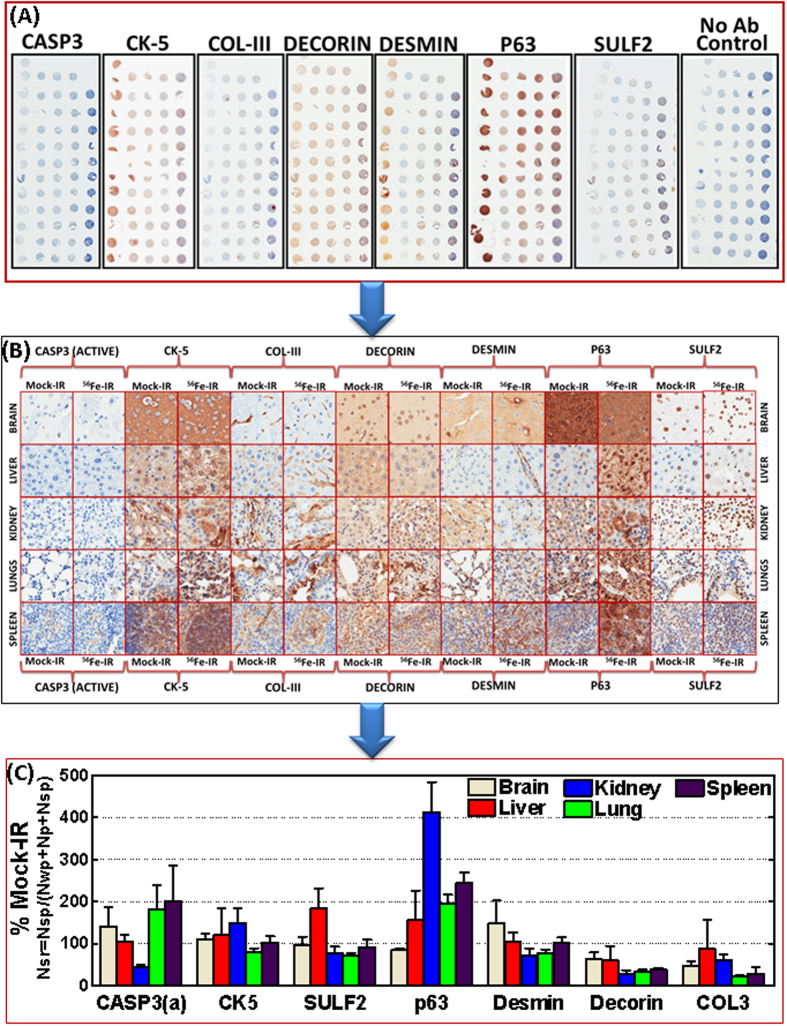
TMA construction coupled with automated IHC analysis showing HZE particle radiation-associated alterations in activated Casp3, CK-5, ColIII, Decorin, Desmin, p63, and SULF2 in brain, kidney, liver, lung, and spleen tissues. (**A**) Representative microphotographs of the TMA constructed with brain, kidney, liver, lung, and spleen tissues from mice exposed to mock-IR or HZE particle radiation, and subjected to automated IHC for Casp3, CK-5, ColIII, Decorin, Desmin, p63, and SULF2. (**B**) Representative photo-micrographs showing random regions (20X) of corresponding tissues exhibiting staining pattern, localization, and the positivity intensity of Casp3, CK-5, ColIII, Decorin, Desmin, p63, and SULF2 after HZE particle radiation compared with mock-IR. (**C**) Histograms obtained from Aperio TMA image quantification analysis showing HZE particle radiation-associated magnifications of Casp3, CK-5, ColIII, Decorin, Desmin, p63, and SULF2 in brain, kidney, liver, lung, and spleen tissues.

To further substantiate that high-LET radiation increases the crucial enzyme, Caspase 3 (apoptosis-related cysteine peptidase), in apoptosis, we examined the cellular expression level of the active form of Caspase 3, **CASP3a**, in brain, liver, kidney, lung, spleen, and gut tissues (Fig. 6A). CASP3a IHC staining revealed a basal level of positivity in mock-irradiated controls, with relatively high expression in spleen and minimal-to-no expression in brain tissues (Fig. 6B). The positive staining of CASP3a protein was predominantly localized in the cytoplasm, often only in a fraction of the cells. Compared with the mock-irradiated controls, CASP3a immunoreactivity was intense (≥2 Fold) in lungs and spleens from the mice exposed to high-LET radiation (Fig. 6B and C). These results were consistent with our TUNEL staining outcomes.

#### (ii) HZE particle radiation modulates early epithelial differentiation proteins CK5 and p63

Tumor protein p63 (**p63**) and Cytokeratin 5 (**CK5**), which are expressed early in epithelial differentiation, have been employed in diagnostic pathology as markers for several types of human neoplasms^35^. The role of p63, including its causal role, in carcinogenesis has been extensively investigated^35^,^36^. Likewise, studies have shown that the transcriptional block-associated non-expression of CK-5 protein can be used as a marker for early cancer diagnosis^37^. To define whether high-LET radiation modulates p63 and CK5, we examined their cellular expression level in brain, liver, kidney, lung, spleen, and gut tissues (Fig. 6A). CK5 IHC staining revealed a basal level of positivity in mock-irradiated controls, with relatively high expression in spleen and brain tissues (Fig. 6B). CK5 positivity was specifically localized to the cytoplasm and cell membrane. CK5 immunoreactivity remained largely unaltered in all tissues investigated. While there was a marginal increase in CK5 expression in kidney tissues, lung tissues exhibited a marginal decrease (Fig. 6C). The tumor protein, p63 IHC staining revealed specific nuclear localization with high baseline expression in mouse brain, kidney, and lungs, while minimal basal expression was observed in liver and spleen tissues (Fig. 6B). Compared with mock-IR controls, high-LET radiation profoundly activated p63 in all tissues investigated, except brain tissues (Fig. 6B and C). We observed a radiation-induced maximal p63 expression (>4 Fold) in kidney tissues (Fig. 6C).

#### (iii) High-LET radiation increases Heparan sulfate endosulfatase, SULF2

SULF2, a neutral PH sulfatase, regulates heparan sulfate proteoglycan (HSPGs) functions by removing 6*-O*-sulfation (6 S) from intact HS-chains, thereby modulating several signaling pathways leading to carcinogenesis. Numerous studies have linked SULF2 activation to hepatocellular, breast, pancreatic, and lung carcinogenesis^38^,^39^,^40^. Researchers have also defined the transformation of non-malignant bronchial epithelial cells with forced Sulf-2 expression^40^. In the present study, SULF2 IHC staining exhibited a strong baseline levels in all tissues investigated (Fig. 6A & B). Compared with tissues from mock-IR mice, high-LET radiation robustly induced SULF2 expression selectively in kidney tissues, while there was no induction of SULF2 in other tissues examined (Fig. 6C).

#### (iv) High-LET radiation completely suppresses matrix proteins Decorin, Collagen III, and Desmin in healthy tissues

Decorin, a ubiquitously expressed proteoglycan, is observed in the stroma of numerous cancers and is protected from the matrix^41^. More importantly, decorin is a mesenchyme-specific gene product and has a paracrine effect on endothelial and epithelial cells. Functionally, soluble and matrix-bound decorin modulates several biological processes, including collagen fibrillogenesis, wound healing, myogenesis, bone physiology, stem cell biology, immunity, angiogenesis, and fibrosis^42^. Researchers have identified that while genetic ablation of decorin permits tumor growth^43^, forced expression of decorin inhibits tumor growth^42^,^44^. Decorin IHC exhibited strong positivity in the extracellular matrix and weak-to-moderate cytoplasmic localization (Fig. 6A). We observed moderate-to-strong baseline levels of Decorin in all tissues investigated (Fig. 6B). Compared with the mock-IR controls, HZE particle radiation resulted in complete and significant suppression of Decorin in all tissues, with maximal inhibition in kidney tissues (Fig. 6C). Likewise, collagens, particularly type III (COL3), a major constituent of the extracellular matrix, highly limit the cancer-spreading ability^45^. We observed a minimum (brain) to strong (lung) positivity in mock-IR controls (Fig. 6A and B). Radiation exposure completely inhibited Col3 localization in brain, kidney, lung, and spleen tissues, with maximal inhibition in lung tissue (Fig. 6C).

Desmin, originally a smooth-muscle type intermediate filament protein, is expressed in peritumoral fibroblasts, reactive (desmoplastic) stroma, cancer- or tumor-associated fibroblasts, and myofibroblasts. Desmin has also been described as a marker of pericytes found in association with blood vessels from the earliest stages of capillary sprouting and throughout angiogenesis. To that end, several studies demonstrated a preferential loss of Desmin in surrounding cells to dysplastic (precancerous) sites and carcinoma. IHC staining revealed selective cytoplasmic localization (Fig. 6A). In most of the tissues investigated, we observed a moderate desmin immunostaining, whereas minimal immunoreaction was seen in liver tissues (Fig. 6B). Though high-LET radiation resulted in a marginal increase in Desmin expression in brain tissues, we observed a considerable Desmin inhibition in kidney and lung tissues (Fig. 6C). Liver and spleen tissues showed no measurable alterations with radiation exposure compared with mock-irradiation.

#### (v) HZE particle radiation promotes and increases micronuclei in healthy tissues

**Figure 7:**
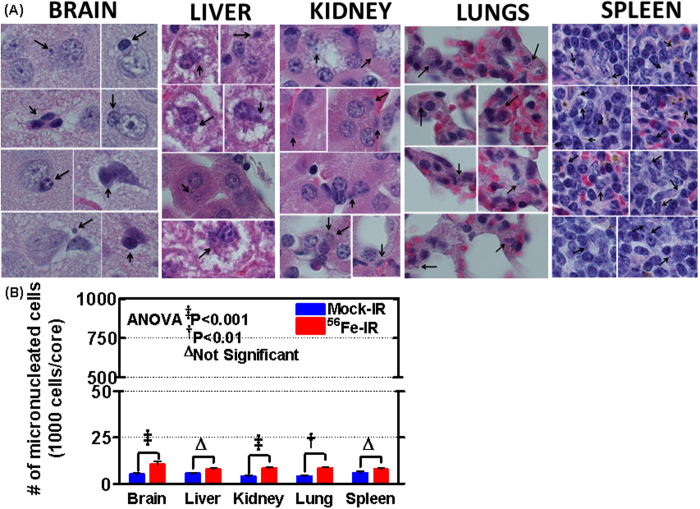
HZE particle radiation-associated increases in micro-nucleated cells. (**A**) Representative microphotographs showing micro-nucleated cells in the brain, kidney, liver, lung, and spleen tissues from mice exposed to HZE particle radiation. Arrowheads point to the micronucleus in each region. (**B**) Histograms obtained from the double-blinded quantification of micro-nucleated cells from brain, kidney, liver, lung, and spleen tissues from mock-IR and HZE particle radiation-exposed mice, showing an HZE-associated significant increase in micronuclei in brain, kidney, and lung tissues.

Micronuclei are widely used as the biomarker for elevated risk in mammalian cells, cultured/exfoliated cells, and biopsy samples^46^. Micronuclei are a reflection of clastogenic events and indicate the ongoing process of DNA damage. This biomarker been correlated with cancer risk at several sites, and its use has been shown to be economical. In addition, micronuclei assay has been used as a biological dosimeter of *in vivo* ionizing radiation exposure^47^,^48^,^49^. In the current study, the frequency of micro-nucleated cells was uniformly elevated in all tissues (brain, liver, kidney, lung, and spleen) from the mice exposed to HZE particle radiation (Fig. 7A and B). Double-blinded scoring of micronuclei quantification revealed a significant increase in micronuclei in brain (*P* < 0.001), kidney (*P* < 0.001), and lung (*P* < 0.01) tissues (Fig. 7B). An increased number of micro-nucleated cells suggest a strong biological effect on chromosomes and chromatid fragments, which responded dramatically to high-LET radiation.

## Discussion

Whether exposure to charged-particle radiation and the levels to which the space travelers are believed to be continuously exposed can elicit potential carcinogenic effects during deep space missions remains a major concern. Since there is increased interest in using particle therapy for cancer treatment, and it is important to understand the delayed carcinogenic effects of heavy ions in deep-space missions, several *in vitro* cell culture models were rapidly developed^50^. Similarly, several genetically modified or tumor-prone animal models were used to examine the possibilities of cancer phenotypes after exposure to charged particle radiation^13^,^51^,^52^. Although the knowledge obtained provided valuable insights, the earlier findings further emphasized the lack of reliability of this data to reflect the response of healthy individuals. Studying tumor incidence after low dose and dose rate of particle radiation in normal, healthy wild-type animal models may warrant the lifetime of the species studied due to prolonged latency. Since molecular and genetic studies may contribute to risk-projection models^53^, ascertaining their altered basic biological processes may be considered an alternative with which to address the concerns of obtaining data in normal healthy tissue within a reasonable time frame.

The present study identified the tissue-specific molecular blueprint that could mediate radiogenic cell transformation and address the concerns of protracted carcinogenic effects of high-LET space radiation in healthy individuals. This response of tissue-specific molecular blueprint was found to be relatively distinctive from the low LET radiation response at similar dose equivalence. The differences are not only in the overall gene expression pattern in a given tissue, but also the changes are less correlative between tissues. Although it is yet to be studied the outcome of the phenotype in response to the differences in the gene expression, LET-dependent differences that were re-validated recently in wild type C57BL/6 mice at 18 months post-irradiation clearly indicated that high LET iron ion tend to develop higher incidence of lung tumor compared to low LET x-rays^16^.

We examined the alterations of oncotargets in tissues of major organs from wild-type C57BL/6 mice in order to closely compare the findings with normal tissue toxicity during cancer treatment and relate the outcomes to the health risks for normal healthy astronauts. It is equally important to understand the prolonged effect of high-LET radiation for radiation risk estimation, since studies of the long-term effects may yield significantly different outcomes of different levels of magnitude than those derived from the short-term cell culture mechanistic studies^54^. The observed inverse dose rate effect in *in vitro* cell culture systems emphasized that the initiation of carcinogensis may arise from entirely different mechanism and may be the product of a short- term effect. This was supported by a study reporting on protracted effects of radiation, comparing one- and twelve-year intervals^54^. We determined the expression of oncotargets at day 30 post-irradiation. Unlike tumor incidence studies which require lifetime observation, studying oncotarget activation in healthy tissue when sustained as long as 30 days could confirm the expected protracted carcinogenic effects in these tissues.

For the first time, we identified the tissue-dependent and –independent oncogenic changes after heavy ion particle radiation. A select group of corrupted cellular genes called oncogenes, which confiscate enable the cells to survive indefinitely and proliferate aberrantly, appeared to orchestrate cancer initiation^55^. When several cancers require altered expression of those sets of non-randomly selected oncogenes and are eventually dependent on those sets of genes to sustain transformed features and progression, this phenomenon is called ‘oncogene addiction’.

Oncogene addiction is a consequence of the fact that the multistage process of carcinogenesis is not simply a summation of the individual effects of activation of multiple oncogenes. Rather, the proteins encoded by these oncogenes will have multiple roles in complex and interacting networks, and their function is also influenced by their activity levels and the context in which they are expressed. More importantly, throughout the multistage carcinogenic process, the evolving mammalian cell must maintain a state of homeostasis between positive-acting and negative-acting factors in order to maintain structural integrity, viability, and the ability to replicate. In addition, it has been postulated that under circumstances a given oncogene may play a more essential and qualitatively different role in a given pathway or ‘module’ compared with its role in normal cells, and orchestrate carcinogenesis^56^. The oncogene addiction model, molecular basis, influencing factors, and clinical implications were first reported by Weinstein in 2002^17^ and have been extensively reviewed in detail in follow up reports^18^,^19^,^57^,^58^, However, the significance of the oncogene addiction concept in relation to radiogenic tumor incidence has not yet been examined. The re-programmed mechanisms that impart HZE particle-driven oncogene(s) addiction by which mammalian cells orchestrate the tumor initiation are largely unknown.

Although it is practically difficult and beyond the scope of this manuscript to discuss the defined role of each of these oncogenes in the initiation of carcinogenesis, it is pertinent to mention that all of the candidates identified here have been extensively investigated in this context. Hence, these oncogenes are archived in the current study’s profile. Here, we restrict our discussion to the crucial carcinogenic players for which we validated the carryover of the radio-responsive transcriptional modulation to the corresponding protein translation.

In the present study, iron ion particle radiation resulted in the heightened transcription of gene *TAL2* in seven out of 8 tissues investigated, which represents a near tissue-independent expression. The observation of the same oncogenes activated in different tissues foster the concept of oncogene addiction. Continued activation or repressions of those specific sets of genes are required for tumor initiation and progression, since the tumors develop a tight dependence on the activation of those genes. *TAL2, a* transcription factor of the basic helix-loop-helix family, was activated at the junctions of chromosomal translocations associated with T-cell acute lymphoblastic leukemia causing overexpression in the T-cell lineage, identifying *TAL2* as an oncogenic transcription factor of T-ALL^59^,^60^. Although *TAL2* has been shown to play a crucial role in brain development^61^, recent investigations have implicated its roles in tumorigenicity and poor prognosis in many tumor systems^62^,^63^. New to science, in the present study, we not only identified that *TAL2* is activated in response to radiation, and HZE particle radiation in particular, but also report that *TAL2* is one potential candidate that is activated in most of the tissues examined including brain, kidney, liver, lung, large and small intestine, and spleen tissues. In brain tissue, after high LET radiation, the *TAL2* expression level was as high as five-fold increase. Interestingly, the comparative QPCR analysis of *TAL 2* expression showed an opposite response with low LET radiation exposure. There is a complete inhibition of *TAL 2* expression in the brain tissue of the normal mice exposed to 2 Gy γ-radiation. Expression levels of *TAL2* after low LET radiation in other tissues such as spleen is similar to the high LET radiation exposure. Albeit, the results further confirm that the gene expression pattern and the severity of the phenotypic outcome between high and low LET radiation exposures are by far distinct from one another, those differences are highly tissue dependent. The pathophysiological consequences due to those tissue-dependent differences in gene expression in high versus low LET radiation exposures are currently not apparent. These findings warrants further studies to ascertain whether LET dependent gene expression play different roles in different tissues.

The activation of the oncogenic addiction pathway is not always restricted to single genes^18^, as in most tumors, oncogenic addiction is dependent on a set of complementing addictive genes. Accordingly, we found that ^56^Fe ion exposure activated an archive of oncogenes transcriptional responses that are faithfully associated with their functional protein translation. This was clearly evident when we investigated crucial molecules that are known to be involved in oncogenic addiction and exhibited different degrees of tissue-specific transcriptional responses, including FOSb and MYCN, which were activated in five tissues; JUNC, JUND, MYB and STAT3, which were activated in four tissues; VEGF-α, which was activated in three tissues, and; cMYC, which was activated in two tissues. Induced levels and activity of JUNC, FOSB, and JUND, the components of AP-1 transcription factor, play a role in the cellular defense against genotoxic stress and, further, have unprecedented influence over transformation and carcinogenesis.

Likewise, of the seven members of the STAT protein family, STAT3 has been shown to play a critical role in carcinogenesis by transducing signals from numerous receptor and non-receptor tyrosine kinases in response to genotoxic stress. STAT3 also regulates the expression of a wide range of genes that contribute to carcinogenesis. Many researchers reported the modulation of these molecules in response to radiation exposure in a manifold of tissue systems and defined the association of these molecules in oncogenic addiction processes leading to transformation and carcinogenesis^64^. In support of that notion, recent studies have shown that high-LET radiation facilitates the stimulation of oncoproteins (JUNC, STAT3, cMYC) and is correlated with the progressive nature of the neoplastic process, at least in breast and liver systems^65^,^66^.

Moreover, VEGFα, the most important ligand for angiogenic processes, has high relevance in oncology and cancer biology by driving increased vascular endothelial cell survival, tube formation/sprouting of new vessels, endothelial migration, and dissolution of the extracellular milieu. The criticality of VEGF-α to carcinogenesis has been reviewed in detail elsewhere^67^. Photons, such as gamma rays, have been shown to increase VEGF-α and subsequent tumor initiation and/or progression^68^,^69^. Likewise, high-LET radiation such as carbon ion radiation has been shown to induce transcription and translation of VEGF-α in cancer cells. To our knowledge, this is the first observation demonstrating the tissue-specific transcriptional and translational alterations of VEGF-α after HZE particle radiation. Further, the activation of oncoproteins (JUNC, JUND, STAT3, cMYC, MYCN, FOSB, MYB, and VEGF-α) in healthy tissues after HZE particle radiation observed here reiterates their crucial role in cellular transformation in response to genotoxic high-LET radiation. However, for the first time, the results presented here identified that this increase in oncoproteins in response to HZE particle radiation is not homogenous, but exhibits tissue-type-specific expression.

Apoptosis is generally considered an anti-carcinogenic process due to its crucial role in eliminating genetically unstable cells that have suffered DNA damage^28^,^29^. Conversely, there is a growing recognition that apoptosis is involved in carcinogenesis through apoptosis-promoting oncogenes^30^,^31^,^32^ that, when coupled with their hyper-proliferative activities, could create a selection pressure to overcome apoptosis. This process would allow cancer cells to acquire a more aggressive malignant phenotype^33^. To that end, researchers have reported that caspase-3, a central effector of apoptosis, facilitates rather than suppressing chemical/radiation-induced genetic instability and carcinogenesis. It has been shown that a significant fraction of cells exposed to radiation can survive Casp3 activation; this sub-lethal activation of Casp3 promoted persistent DNA damage and oncogenic transformation^34^. In the present study, we observed a statistically insignificant, marginal increase in apoptosis, as measured with TUNEL assay. More importantly, for the first time, we demonstrated the defined sub-lethal level activation of Casp3 that could correspond to the HZE particle radiation-associated genetic instability and carcinogenesis.

As a measure of carcinogenic events, we identified HZE particle radiation-associated changes in CK5 and p63. These early epithelial differentiation proteins have been employed in diagnostic pathology as markers for several types of human neoplasms^35^. The causal role of p63 in carcinogenesis has been extensively investigated^35^,^36^. Studies have also shown that the transcriptional block-associated non-expression of CK5 can be used as a marker for early cancer diagnosis^37^. In the present study, we revealed that HZE particle radiation exposure resulted in the unaltered expression of CK5 and profound activation of p63 in healthy tissues, neither of which has been reported earlier.

Further, we identified that HZE particle radiation induced SULF2 selectively in kidney tissues. SULF2 regulates Heparan sulfate proteoglycan (HSPGs) functions by removing 6*-O*-sulfation (6S) from intact HS-chains, thereby modulating several signaling pathways leading to carcinogenesis. Numerous independent studies have causally linked SULF2 activation to hepatocellular, breast, pancreatic, and lung carcinogenesis^38^,^39^,^40^. Researchers have also demonstrated transformation of non-malignant epithelial cells with forced Sulf-2 expression^40^. To our knowledge, this is the first study identifying the kidney-specific activation of SULF2 after HZE radiation.

Further substantiating the carcinogenic effects of HZE radiation, we demonstrated the regulation of matrix proteins Decorin, Collagen III, and Desmin. Decorin, a ubiquitously expressed proteoglycan, is expressed in the stroma of a manifold of cancers and is protected from the matrix^41^. More importantly, decorin is a mesenchyme-specific gene product and has a paracrine effect on endothelial and epithelial cells. Functionally, soluble and matrix-bound decorin modulate several biological processes, including collagen fibrillogenesis, wound healing, myogenesis, bone physiology, stem cell biology, immunity, angiogenesis, and fibrosis^42^.

Researchers have identified that while genetic ablation of decorin permits tumor growth^43^, forced expression of decorin inhibits tumor growth^42^,^44^. Likewise, collagens, particularly type III (COL3), a major constituent of the extracellular matrix, limit the ability of cancer cells to spread^45^. In addition, desmin, originally a smooth-muscle type intermediate filament protein, is expressed in peritumoral fibroblasts, reactive (desmoplastic) stroma, cancer- or tumor-associated fibroblasts, and myofibroblasts. Desmin has also been described as a marker of pericytes found in association with blood vessels from the earliest stages of capillary sprouting and throughout angiogenesis. Consistently, numerous studies demonstrated a preferential loss of Desmin in surrounding cells to dysplastic (precancerous) sites and carcinoma. In conceptual accordance with these findings, here we demonstrated that HZE iron ion radiation resulted in a measurable, yet tissue-specific, decrease in decorin, Col3, and Desmin.

Oncogene addiction theory brought up the possibility of involvement of non-oncogene addiction pathways in tumorigenesis^70^. A remarkable tumor response to the approach of targeting a single mediator has been noted in drug response studies. The targets that are not classical oncogene- driven oncoproteins, but upon which tumors rely for initiation and maintenance, are referred to as ‘non-oncogene addiction’. Heat shock factor-1 (HSF-1) has been shown to be one of the non-oncogene factors whose absence negates transformation induced by oncogenic H-RAS^V12D^ or PDGF-B, but its overexpression was unable to transform immortalized mouse embryonic fibroblasts^71^. It is, therefore, possible that radiation exposure may simultaneously impart non-oncogene addiction through inducible stress responsive factors, including extracellular-matrix remodeling, oxidative stress, and DNA damage. For example, successive generation with the transmissible DNA damage produced by radiation exposure could cause genomic instability.

Sustained genomic instability and its association with carcinogenesis are well established. Though genomic instability is seldom observed in transformed cells, the existence of genomic instability is clearly an indicative marker for an increased neoplastic transforming response to radiation. Pro-tumorigenic cellular models have shown that 1 GeV/nucleon ^56^Fe ion can lead to transformation process in pro-tumorigenic lung cells after successive generation with accumulating genetic instability^72^. To estimate the elevated risk of acquiring genomic instability in mammalian cells, cultured/exfoliated cells, and biopsy samples, the existence of micronuclei is widely used as the standard biomarker^46^. Thus, micronuclei assay has also been used as a biological dosimeter of *in vivo* ionizing radiation exposure^47^,^48^,^49^, since it reflects clastogenic events and indicates the ongoing process of cellular DNA damage. In order to follow the non-oncogenic pathways that instigate the carcinogenic process after HZE particle radiation, in the current study, we examined the existence of micronuclei in healthy tissues *in vivo*. The increased number of micro-nucleated cells observed here suggested a strong biological effect on chromosomes and chromatid fragments, which responded dramatically to high-LET radiation, and were consistent with the outcomes of the other molecular endpoints investigated.

Earlier studies^73^ emphasized that protracted carcinogenic events in normal tissue after HZE ion radiation may progress through a two-stage clonal expansion model^73^. The data we obtained on simultaneous activation of oncotargets that are involved in both oncogene addiction and non-oncogene addiction signaling pathways underscore the two-stage clonal expansion theory. Our data further reveals additional novel concept that the two-phase clonal expansion may be driven concurrently rather than consecutively. It can be rationalized that while the oncogenic addiction signaling governs the radiation-induced initiation of new damage in normal tissue, triggering of the non-oncogene addiction pathway may concurrently prepare those cells that are already initiate to tumor progression pathway.

In conclusion, the results of the present study identified a tissue-specific molecular blueprint pertaining to oncogene addiction that could mediate radiogenic cell transformation after high-LET space radiation. Tissue scoring identified that the *TAL2* oncogene may serve as a potential target for measuring HZE particle radiation-induced carcinogenesis in most tissues. More importantly, HZE particle radiation-associated oncogene related transcriptional modulations were shown to correspond to the protein modulations in healthy tissues as evidenced and validated by the expression of crucial oncoproteins, including TAL2, FOSb, MYCN, JUNC, JUND, MYB, STAT3, VEGFα, and cMYC, in the manifold of healthy tissues. HZE particle radiation-induced initiation of carcinogenesis is consistent with the observed alterations in sub-lethal levels of active Casp3; induction of Heparan sulfate endosulfatase SULF2; regulation of matrix proteins Decorin, Collagen III and Desmin, and; increase in tissue-specific micro-nucleated cells. The data presented here define, for the first time, the tissue-specific oncogenic molecular blueprint and carcinogenic process in response to HZE particle radiation. In parallel, the contribution of bystander or non-targeted effects, which have been extensively discussed in recent years, in the initiation and sustaining the progression of the transformed clones should be strongly considered. Combining the influence of bystander mechanisms in the context of oncogene and non-oncogene addiction pathways, although complex, will provide valuable insights to develop reasonable counter measures.

The present study not only reports several new findings, but also provides a platform for further investigation into different radiation quality, dose/dose rate effect, and linear energy transfer (LET) in healthy tissue. For example our observation on comparing the microarray profiling of high LET versus low LET radiation exposure of tissues from normal wild type animals revealed a differential pattern, which further underscores the possible existence of a unique molecular signature depending upon the quality of radiation. This work be expanded further in several other viable preclinical models to define the importance of age, gender, tissue specificity and tumor cell heterogeneity, whilst determining the specific nature of oncogene activation, radiogenic cell transformation and carcinogenesis in normal animals.

## Additional Information

**How to cite this article**: Aravindan, N. *et al*. High Energy Particle Radiation-associated Oncogenic Transformation in Normal Mice: Insight into the Connection between Activation of Oncotargets and Oncogene Addiction. *Sci. Rep.* **6**, 37623; doi: 10.1038/srep37623 (2016).

**Publisher’s note:** Springer Nature remains neutral with regard to jurisdictional claims in published maps and institutional affiliations.

## Supplementary Material

Supplementary Table S1

Supplementary Figure S1

Supplementary Figure S2
