# Supplementary material for: High Energy Particle Radiation-associated Oncogenic Transformation in Normal Mice: Insight into the Connection between Activation of Oncotargets and Oncogene Addiction
Source: Sci Rep. 2016 Nov 23;6:37623. doi: 10.1038/srep37623 (PMC5120307; doi:10.1038/srep37623)
Supplement: Supplementary Table S1 [file srep37623-s1.doc]

**High Energy Particle Radiation-associated Oncogenic Transformation in Normal Mice: Insight into the Connection between Activation of Oncotargets and Oncogene Addiction**

Natarajan Aravindan^1^, Sheeja Aravindan^2^, Krishnan Manickam^3^ and Mohan Natarajan^3^

| **Table S1:** List of oncogenes archived in the QPCR profiling, and their location and types. | |  |  |
| --- | --- | --- | --- |
|  |  |  |  |
| **Symbol** | **Entrez Gene Name** | **Location** | **Type(s)** |
| ABL1 | ABL proto-oncogene 1, non-receptor tyrosine kinase | Nucleus | kinase |
| ABL2 | ABL proto-oncogene 2, non-receptor tyrosine kinase | Cytoplasm | kinase |
| AKAP13 | A kinase (PRKA) anchor protein 13 | Cytoplasm | other |
| AKT2 | v-akt murine thymoma viral oncogene homolog 2 | Cytoplasm | kinase |
| ALK | anaplastic lymphoma receptor tyrosine kinase | Plasma Membrane | kinase |
| AXL | AXL receptor tyrosine kinase | Plasma Membrane | kinase |
| BCL2 | B-cell CLL/lymphoma 2 | Cytoplasm | transporter |
| BCL3 | B-cell CLL/lymphoma 3 | Nucleus | transcription regulator |
| BCL6 | B-cell CLL/lymphoma 6 | Nucleus | transcription regulator |
| BRCA1 | breast cancer 1, early onset | Nucleus | transcription regulator |
| BRCA2 | breast cancer 2, early onset | Nucleus | transcription regulator |
| CCND1 | cyclin D1 | Nucleus | transcription regulator |
| CSF1R | colony stimulating factor 1 receptor | Plasma Membrane | kinase |
| E2F1 | E2F transcription factor 1 | Nucleus | transcription regulator |
| E2F3 | E2F transcription factor 3 | Nucleus | transcription regulator |
| EGFR | epidermal growth factor receptor | Plasma Membrane | kinase |
| ELK1 | ELK1, member of ETS oncogene family | Nucleus | transcription regulator |
| ERBB2 | erb-b2 receptor tyrosine kinase 2 | Plasma Membrane | kinase |
| ERBB3 | erb-b2 receptor tyrosine kinase 3 | Plasma Membrane | kinase |
| ERBB4 | erb-b2 receptor tyrosine kinase 4 | Plasma Membrane | kinase |
| FES | FES proto-oncogene, tyrosine kinase | Cytoplasm | kinase |
| FGF4 | fibroblast growth factor 4 | Extracellular Space | growth factor |
| FGFR2 | fibroblast growth factor receptor 2 | Plasma Membrane | kinase |
| FGR | FGR proto-oncogene, Src family tyrosine kinase | Nucleus | kinase |
| FOS | FBJ murine osteosarcoma viral oncogene homolog | Nucleus | transcription regulator |
| FOSB | FBJ murine osteosarcoma viral oncogene homolog B | Nucleus | transcription regulator |
| FOSL1 | FOS-like antigen 1 | Nucleus | transcription regulator |
| FOSL2 | FOS-like antigen 2 | Nucleus | transcription regulator |
| FOXO1 | forkhead box O1 | Nucleus | transcription regulator |
| GIP | gastric inhibitory polypeptide | Extracellular Space | other |
| GLI1 | GLI family zinc finger 1 | Nucleus | transcription regulator |
| GTF2H1 | general transcription factor IIH, polypeptide 1, 62kDa | Nucleus | transcription regulator |
| HGF | hepatocyte growth factor (hepapoietin A; scatter factor) | Extracellular Space | growth factor |
| HRAS | Harvey rat sarcoma viral oncogene homolog | Plasma Membrane | enzyme |
| JUN | jun proto-oncogene | Nucleus | transcription regulator |
| JUNB | jun B proto-oncogene | Nucleus | transcription regulator |
| JUND | jun D proto-oncogene | Nucleus | transcription regulator |
| KIT | v-kit Hardy-Zuckerman 4 feline sarcoma viral oncogene homolog | Plasma Membrane | transmembrane receptor |
| KMT2A | lysine (K)-specific methyltransferase 2A | Nucleus | transcription regulator |
| KRAS | Kirsten rat sarcoma viral oncogene homolog | Cytoplasm | enzyme |
| LCK | LCK proto-oncogene, Src family tyrosine kinase | Cytoplasm | kinase |
| LMO1 | LIM domain only 1 (rhombotin 1) | Nucleus | transcription regulator |
| LMO2 | LIM domain only 2 (rhombotin-like 1) | Nucleus | transcription regulator |
| LYL1 | lymphoblastic leukemia associated hematopoiesis regulator 1 | Nucleus | transcription regulator |
| LYN | LYN proto-oncogene, Src family tyrosine kinase | Cytoplasm | kinase |
| MAS1 | MAS1 proto-oncogene, G protein-coupled receptor | Plasma Membrane | G-protein coupled receptor |
| MCF2 | MCF.2 cell line derived transforming sequence | Cytoplasm | other |
| MCF2L | MCF.2 cell line derived transforming sequence-like | Cytoplasm | other |
| MDM2 | MDM2 proto-oncogene, E3 ubiquitin protein ligase | Nucleus | transcription regulator |
| MET | MET proto-oncogene, receptor tyrosine kinase | Plasma Membrane | kinase |
| MLH1 | mutL homolog 1 | Nucleus | enzyme |
| MOS | v-mos Moloney murine sarcoma viral oncogene homolog | Cytoplasm | kinase |
| MSH2 | mutS homolog 2 | Nucleus | enzyme |
| MYB | v-myb avian myeloblastosis viral oncogene homolog | Nucleus | transcription regulator |
| MYC | v-myc avian myelocytomatosis viral oncogene homolog | Nucleus | transcription regulator |
| MYCL | v-myc avian myelocytomatosis viral oncogene lung carcinoma derived homolog | Nucleus | transcription regulator |
| MYCN | v-myc avian myelocytomatosis viral oncogene neuroblastoma derived homolog | Nucleus | transcription regulator |
| NFKB1 | nuclear factor of kappa light polypeptide gene enhancer in B-cells 1 | Nucleus | transcription regulator |
| NFKB2 | nuclear factor of kappa light polypeptide gene enhancer in B-cells 2 (p49/p100) | Nucleus | transcription regulator |
| NRAS | neuroblastoma RAS viral (v-ras) oncogene homolog | Plasma Membrane | enzyme |
| Nrg1 | neuregulin 1 | Plasma Membrane | other |
| NTRK1 | neurotrophic tyrosine kinase, receptor, type 1 | Plasma Membrane | kinase |
| PAX5 | paired box 5 | Nucleus | transcription regulator |
| PDGFA | platelet-derived growth factor alpha polypeptide | Extracellular Space | growth factor |
| PDGFB | platelet-derived growth factor beta polypeptide | Extracellular Space | growth factor |
| PIM1 | Pim-1 proto-oncogene, serine/threonine kinase | Cytoplasm | kinase |
| PMS1 | PMS1 homolog 1, mismatch repair system component | Nucleus | enzyme |
| PMS2 | PMS1 homolog 2, mismatch repair system component | Nucleus | enzyme |
| RAF1 | Raf-1 proto-oncogene, serine/threonine kinase | Cytoplasm | kinase |
| RB1 | retinoblastoma 1 | Nucleus | transcription regulator |
| RET | ret proto-oncogene | Plasma Membrane | kinase |
| ROS1 | ROS proto-oncogene 1 , receptor tyrosine kinase | Plasma Membrane | kinase |
| RUNX1 | runt-related transcription factor 1 | Nucleus | transcription regulator |
| SKI | SKI proto-oncogene | Nucleus | transcription regulator |
| SRC | SRC proto-oncogene, non-receptor tyrosine kinase | Cytoplasm | kinase |
| STAT3 | signal transducer and activator of transcription 3 (acute-phase response factor) | Nucleus | transcription regulator |
| STAT5B | signal transducer and activator of transcription 5B | Nucleus | transcription regulator |
| TAL1 | T-cell acute lymphocytic leukemia 1 | Nucleus | transcription regulator |
| TAL2 | T-cell acute lymphocytic leukemia 2 | Other | other |
| TCF7L2 | transcription factor 7-like 2 (T-cell specific, HMG-box) | Nucleus | transcription regulator |
| TFDP2 | transcription factor Dp-2 (E2F dimerization partner 2) | Nucleus | transcription regulator |
| TGFB1 | transforming growth factor, beta 1 | Extracellular Space | growth factor |
| TGFB2 | transforming growth factor, beta 2 | Extracellular Space | growth factor |
| TIAM1 | T-cell lymphoma invasion and metastasis 1 | Cytoplasm | other |
| TLX1 | T-cell leukemia homeobox 1 | Nucleus | transcription regulator |
| TSC2 | tuberous sclerosis 2 | Cytoplasm | other |
| VAV1 | vav 1 guanine nucleotide exchange factor | Nucleus | transcription regulator |
| VEGFA | vascular endothelial growth factor A | Extracellular Space | growth factor |
