# Supplementary material for: High Energy Particle Radiation-associated Oncogenic Transformation in Normal Mice: Insight into the Connection between Activation of Oncotargets and Oncogene Addiction
Source: Sci Rep. 2016 Nov 23;6:37623. doi: 10.1038/srep37623 (PMC5120307; doi:10.1038/srep37623)
Supplement: Supplementary Figure S1 [file srep37623-s2.doc]

**High Energy Particle Radiation-associated Oncogenic Transformation in Normal Mice: Insight into the Connection between Activation of Oncotargets and Oncogene Addiction**

Natarajan Aravindan^1^, Sheeja Aravindan^2^, Krishnan Manickam^3^ and Mohan Natarajan^3^


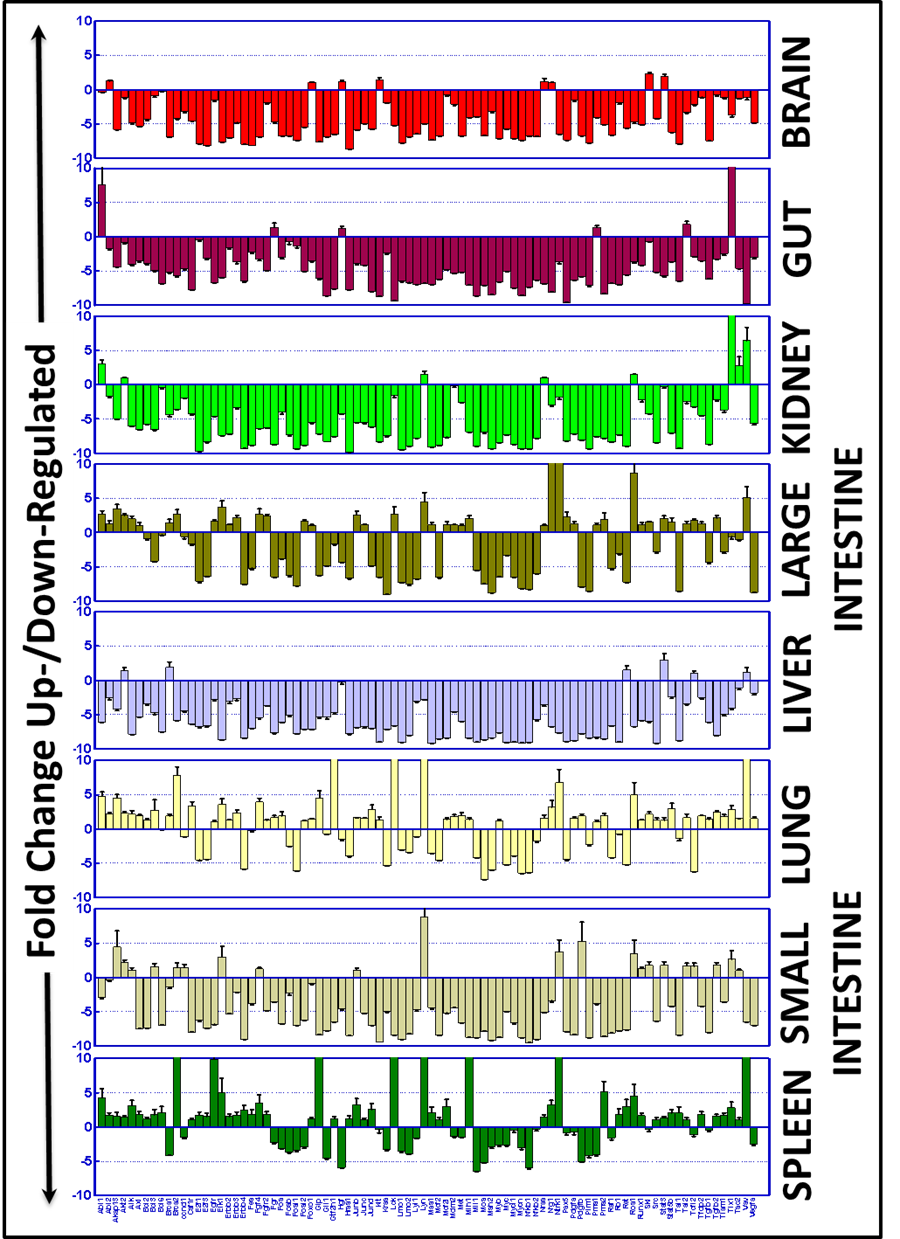


**Figure S1:** Histograms obtained from the QPCR profiling showing transcriptional modifications of 88 oncogenes in mouse brain, gut, kidney, liver, lung, spleen, and large and small intestine in response to low-LET exposure. The relative expression level of each gene is expressed as fold change compared with the mock-IR controls (mean and SD). For ease of comparison, all genes investigated, irrespective of their expression status, are included in the graph.
