# Supplementary material for: High Energy Particle Radiation-associated Oncogenic Transformation in Normal Mice: Insight into the Connection between Activation of Oncotargets and Oncogene Addiction
Source: Sci Rep. 2016 Nov 23;6:37623. doi: 10.1038/srep37623 (PMC5120307; doi:10.1038/srep37623)
Supplement: Supplementary Figure S2 [file srep37623-s3.doc]

**High Energy Particle Radiation-associated Oncogenic Transformation in Normal Mice: Insight into the Connection between Activation of Oncotargets and Oncogene Addiction**

Natarajan Aravindan^1^, Sheeja Aravindan^2^, Krishnan Manickam^3^ and Mohan Natarajan^3^


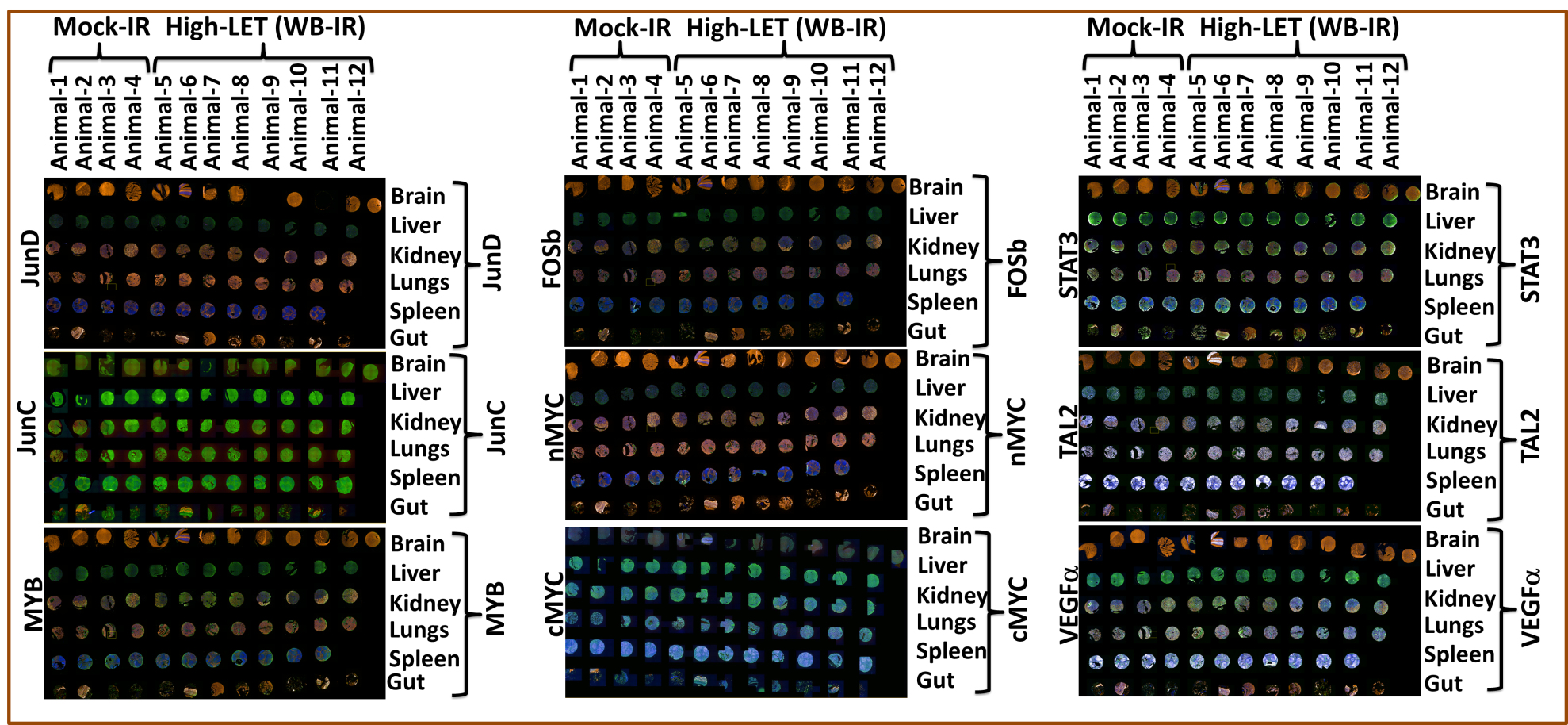


**Figure S2:** Fluorescent microphotographs of tissue microarray (TMA) constructed with brain, liver, kidney, lung, spleen, and gut tissues from mice exposed to mock-irradiation or whole body HZE particle radiation and immunostained for JUND, JUNC, FOSb, MYB, MYCN, cMYC, STAT3, TAL2, and VEGFa. The primary protein was tagged with secondary Ab tagged with Alexa Fluor-488®, while the cell membrane was marked with WGA-Alexa Fluor-594® and nuclear counterstained with DAPI. Stained TMA were then subjected to high content IF confocal imaging.
